# From ultra-processed foods towards healthy eating for CKD patients: a proposal of educational infographics

**DOI:** 10.1007/s40620-023-01817-3

**Published:** 2023-11-23

**Authors:** Marina Padial, Angeline Taylor, Alice Sabatino, Giorgina Barbara Piccoli, Carla Maria Avesani

**Affiliations:** 1 Division of Renal Medicine, Department of Clinical Science, Intervention and Technology, Karolinska Institute, Stockholm, Sweden; 2 Servicio de Endocrinología y Nutrición, Instituto de Investigación Biomédica de Málaga-Plataforma BIONAND, Hospital Regional Universitario de Málaga, Avda. Carlos Haya, s/n, 29010 Málaga, Spain; 3 Departamento de Medicina y Dermatología, Universidad de Málaga, Málaga, Spain; 4 Renal Unit, Royal Devon and Exeter (Wonford) Hospital, Exeter, UK; 5 Department of Nephrology, Parma University Hospital, Parma, Italy; 6 Néphrologie et Dialyse, Centre Hospitalier Le Mans, venue Rubillard, 72037 Le Mans, France

**Keywords:** Ultraprocessed food, Chronic kidney disease, Nutrition education, Dietary pattern

## Abstract

Ultraprocessed food (UPF) is defined as industrialized, packaged and ready-to-eat food produced on a large scale, using sophisticated industrial machinery. Examples of UPFs include salty and sweet snacks, industrialized biscuits and packaged meals, processed meats and sugary drinks. Ultraprocessed food has a long-shelf life, is highly palatable, microbiologically safe, affordable and most of all, easy to consume. For these reasons, its consumption has been increasing worldwide, and is replacing healthy homemade meals. The main concern of this dietary shift is that UPFs come with the addition of salt, sugar, unhealthy fats, and several additives and taste enhancers that contain, among other substances, relevant quantities of potassium, phosphate and sodium. A large proportion of UPF in the diet may carry risks for patients with chronic kidney disease (CKD) since it can worsen blood pressure and glycemic control, and lead to constipation, hyperkalemia and hyperphosphatemia. Acknowledging the importance of UPF in kidney health implies integrating nutritional counseling with information on UPFs, and specific educational material can be helpful for patients, caregivers, and also for health care providers. We developed a set of 3 infographics dedicated to CKD patients, with information on how to identify UPFs, reasons for decreasing consumption, how to compose a healthy CKD plate and tips for reading food labels in supermarkets and grocery shops. We hope that this material can be useful in CKD outpatient clinics and dialysis centers as well as in general practitioners’ offices, caring for early stage CKD.

**Supplementary Information:**

The online version contains supplementary material available at 10.1007/s40620-023-01817-3.

## Background

Dietary patterns have drastically changed since the development of ultra-processed foods (UPFs), with a shift from a regional to a global and industrialized diet. Ultraprocessed foods can be defined as mechanized, packaged, mass produced, ready-to-eat foods, produced using sophisticated industrial machinery [1]. Ultraprocessed foods usually contain food additives and preservatives that can increase shelf-life and change food color and consistency to make it more attractive to consumers. Fats, salt, and sugar are usually added to UPFs to increase palatability and, often also non-caloric sweeteners [1]. Conversely, dietary fiber content is usually poor. Of note, the availability of vegan and vegetarian UPFs to address the needs of those who wish to shift away from animal protein to help the environment is increasing [2, 3]. In addition, supermarkets display shelves with UPFs to satisfy the needs of individuals on specific diets, such as ketogenic, low carb, and low sodium. In the past decade, many studies have shown that a higher proportion of UPF is associated with increased risk of developing obesity and non-communicable diseases [4–6], and more recently, even with cognitive impairment [5, 7]. Consumption of UPFs is high all over the world [8]. Supplementary Table 1 briefly describes the appeal to consumers, explaining the fast increase in sales.

However, UPFs may have many detrimental effects on health [9]. Since UPFs have been gradually replacing healthy foods, namely fruits, vegetables, legumes and nuts, a diet high in UPFs can trigger intestinal constipation, result in a change in gut microbiota, and contribute to the development of inflammatory bowel diseases [10–12]. In chronic kidney disease (CKD), the intake of UPFs by patients is of particular concern. Epidemiological studies performed in large cohorts have consistently suggested that a high UPF intake increases the risk of developing CKD and accelerates CKD progression [13–16]. In kidney transplant recipients, higher UPF consumption has been associated with lower allograft survival and higher morbidity and mortality [17]. Additional concerns for CKD patients include the risk of developing hyperkalemia and hyperphosphatemia due to food additives that contain potassium and phosphate. Passing on this knowledge to patients is necessary and may improve adherence to more healthy dietary habits. We here propose three leaflets attempting to provide practical information about UPFs, healthy diet and food labeling in CKD, developed in a language-friendly manner for patients, caregivers and health care providers. The first leaflet describes the main differences between UPFs and natural foods and reasons for limiting UPFs, the second provides a simple guide on how to interpret food labels, and the third contains practical tips on how to assemble a healthy plate for patients with CKD.

## Ultraprocessed food can impair diet quality and is a hidden source of dietary potassium and phosphate

Traditionally, dietary recommendations to prevent hyperkalemia suggest reducing the intake of healthy sources of potassium, such as fruits, vegetables, legumes, whole grains and nuts [18, 19]. Dairy products and red meat are also sources of potassium. Common recommendations to control hyperphosphatemia encompass restricting mainly animal-derived proteins, such as meat, fish, eggs, and dairy products [20]. Ultimately, these recommendations may lead to a paradox whereby following a low potassium and low phosphorus diet, a healthy diet with fruits, vegetables, legumes and nuts is not possible [21]. Hence, the diet that is recommended to patients with advanced CKD and on dialysis may become low in fiber, restrictive, monotonous and difficult to follow [22, 23]. However, recent studies have shown that the bioavailability of potassium and phosphate from food additives is of the order of 90–100%, while that from natural foods is much lower, around 50–60% [24–26]. Therefore, reducing UPFs should be the first step towards avoiding hyperkalemia and other disorders related to phosphate, such as mineral and bone disorder. Phosphate retention plays an important role in the development of mineral and bone disorder, and reducing phosphate intake can ameliorate its clinical consequences [27]. The risks of food additives, not necessarily linked to CKD are summarized in Table 1.

**Table 1:** Food additives and potentially harmful risks

Category	E-number (Europe) [28, 29]	Potentially harmful risks [30]
Sweeteners	E950–E967, E420–E421 Examples: acesulfame K (E950), aspartame (E951), sorbitols (E420)	Potential carcinogenic effect, allergies, metabolic dysregulation
Dyes	E100–E180 Examples: curcumins (E100), amaranth (E123), titanium dioxide (E171)	Potential carcinogenic effect, allergies, ADHD in children
Preservatives	E200–E297 Examples: potassium sorbate (E202), sodium benzoate (E211), potassium nitrate (E252)	Carcinogenic effects, hypertension, cardiovascular disease
Antioxidants and acidity regulators	E300–E385 Examples: phosphoric acid (E338), potassium phosphates (E340), potassium lactate (E326)	No potential toxic effect, but high doses can worsen existing diseases. This is the case of phosphorus-based additives in CKD
Emulsifiers, stabilizers, and thickeners	E400—E585 Examples: diphosphates (E450), triphosphates (E451), potassium sulfates (E515)	Malabsorption, bowel distention and obstruction, mucous membrane irritation, change in microbiota. Worsening of kidney and liver dysfunctions
Flavor enhancers	E620–E640 Examples: monosodium glutamate (E621), monopotassium glutamate (E622), calcium glutamate (E623)	Allergies and gastrointestinal symptoms

*ADHD* attention deficit hyperactivity disorder, *CKD* chronic kidney disease

**Fig. 1:**
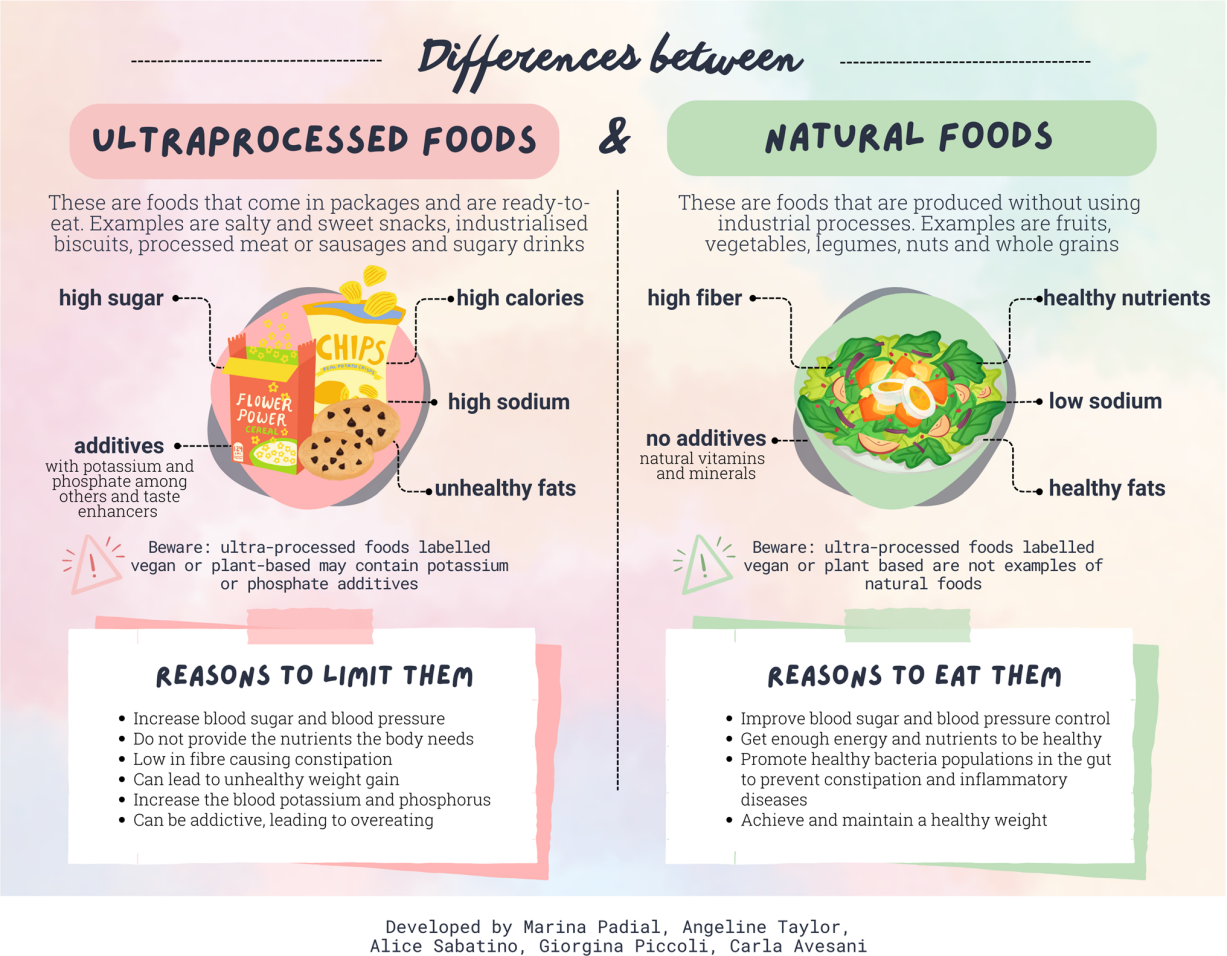
Leaflet with information on how to distinguish ultraprocessed foods (UPFs) from natural foods and with reasons for limiting UPFs in the diet

The fear that fruits, vegetables and nuts can lead to hyperkalemia can increase the risk that patients may replace healthy foods with UPFs, believing that they are lower-potassium options. Therefore, it is important to deliver information on how to identify UPFs, reasons for limiting them, and why patients should consume more fresh foods with only a minimal degree of industrial processing. This is described in the leaflet shown in Fig. 1.

**Fig. 2:**
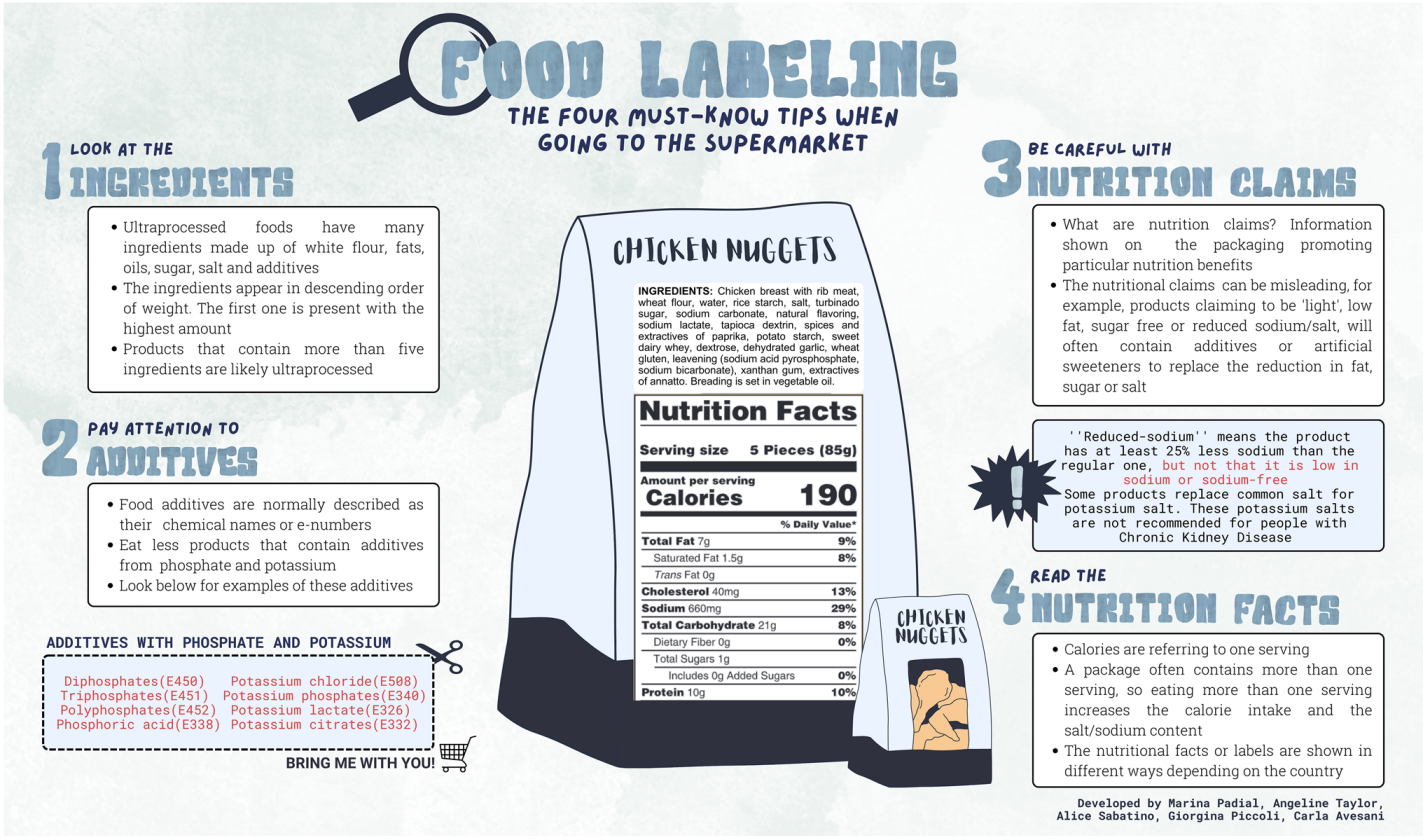
Leaflet with information about food labeling

The second leaflet (Fig. 2) complements the previous one with information on how to identify UPF in food labels. Since food labeling varies considerably from country to country and, often, the information on food additives is neither clear nor easy to understand, this leaflet uses a hypothetical food-label, highlighting how to read ingredients to find out if the product is a UPF, how to pay attention to food additives especially if they contain potassium and phosphate, and how to read the claim “reduced-sodium” replacing sodium with potassium salts. Usually, the label starts by showing the serving size, calories and nutrient content per serving and per 100 g of the product. The macronutrients (fat, carbohydrates and protein) are usually reported first, sometimes with a distinction between different types of fats, and information on fiber and type of carbohydrates. Some labels also add the vitamin content (natural or added). This leaflet can be taken to the supermarket as a simple guide to check the main additives based on potassium and phosphate.

## The priority should be to follow a healthy diet

**Fig. 3:**
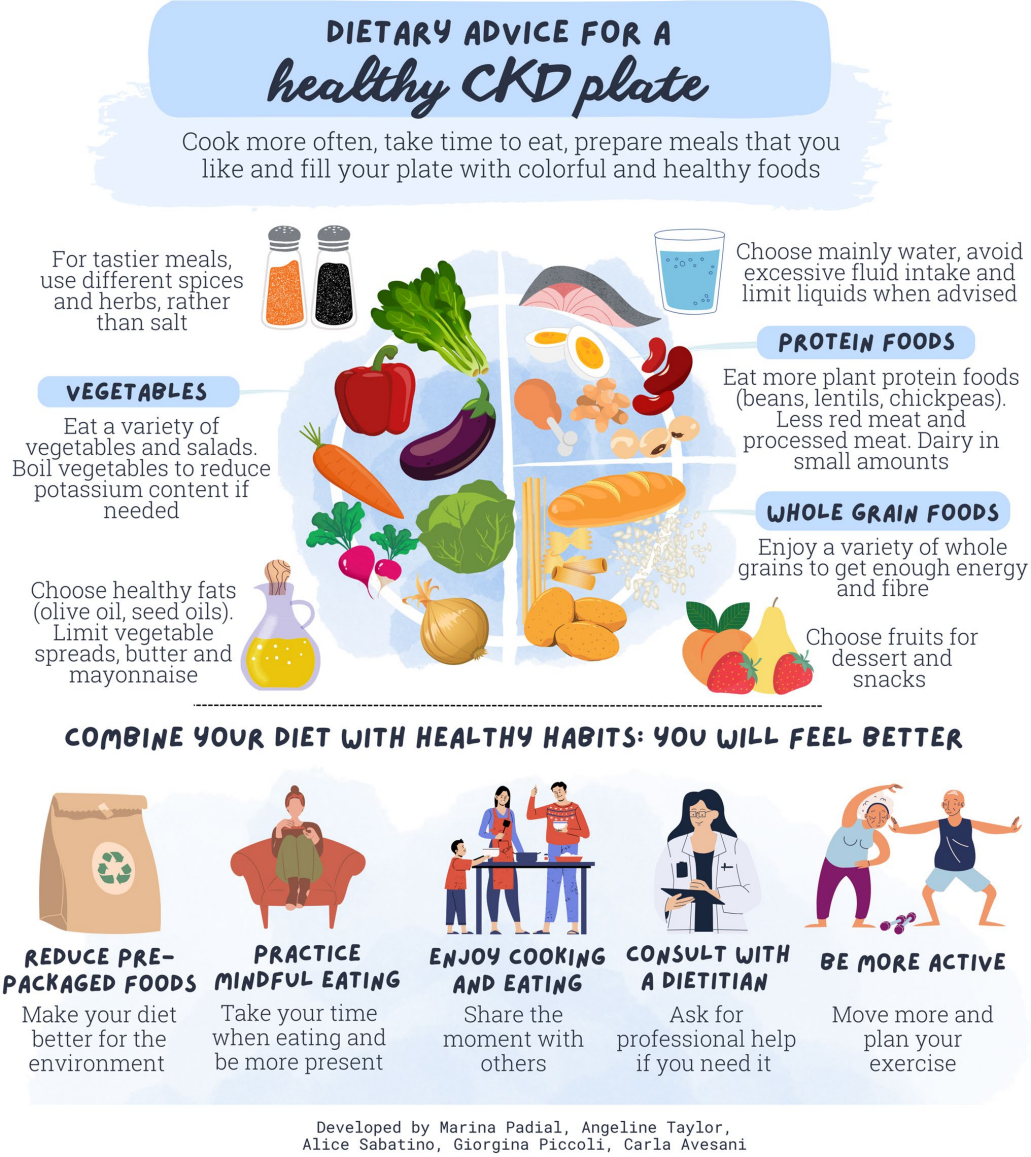
Leaflet with information on how to assemble a healthy CKD food plate

We do not eat calories and nutrients—we eat food. Therefore, it is essential that we communicate with patients using a language that conveys the acquired knowledge into a healthy CKD food plate, which is the focus of the third leaflet (Fig. 3). On the web there are many examples of “renal plates”. Using a Google search (on August 18th, 2023), typing “renal plates” and “CKD plates” we found examples presented in graphics and videos, most of them emphasizing increasing the intake of plant-derived foods. This is in contrast with the frequent dietary advice to reduce fruits and vegetables in patients with advanced CKD or dialysis. However, the quantity, and the energy intake—which is acknowledged as being of vital importance in CKD—is seldom discussed. Moreover, food additives are briefly mentioned without providing details. Of note, some of this material refers to information on packaged food as a useful source of information and suggests “asking a dietitian” about natural foods. Prepared meals for CKD patients are also gaining more attention on the web, and some solutions are quite attractive; however, these often have a relatively high cost ($15–20 USD/meal), but typically with a low energy content (350–500 kcal per meal), and do not contain information on additives and preservative agents, which are very likely needed to allow for delivery of 20-meal packages. Altogether, it is clear that a “Google search” does not provide adequate guidance on how to prepare a healthy CKD plate.

While the healthy CKD plate proposed by the third infographic (Fig. 3) is in keeping with the other educational leaflets, we considered that it is worth adding to emphasize that healthy eating is part of a healthier life-style, from planet consciousness to limiting UPF consumption.

## Conclusions

The intake of UPFs is increasing world-wide, and the authors wish to share educational infographic material designed for CKD patients, their caregivers and healthcare providers as there is increasing need to help patients identify and limit UPF consumption. We have attempted to provide graphic information with reasons for decreasing UPFs in the diet, how to identify UPFs with tips for reading food labels in supermarkets and grocery shops, and on how a healthier diet can be designed for patients with CKD. We hope that this material can be useful in outpatient and dialysis clinics, and shared among carers. The authors are available for adding or developing further information upon patients’ and caregivers’ requests.

### Supplementary Information

Below is the link to the electronic supplementary material.

Supplementary file1 (DOC 40 kb)
