# Supplementary material for: From ultra-processed foods towards healthy eating for CKD patients: a proposal of educational infographics
Source: J Nephrol. 2023 Nov 23;37(2):323–9. doi: 10.1007/s40620-023-01817-3 (PMC11043116; doi:10.1007/s40620-023-01817-3)
Supplement: Supplementary file 1 — Supplementary file1 (DOC 40 kb) [file 40620_2023_1817_MOESM1_ESM.doc]

**Supplementary Table**

**Supplementary Table 1. Characteristics of ultraprocessed food that makes it commercially attractive for consumers**

| - Easy to use, since these are usually presented as ready-to-eat or ready-to-heat foods. - Attractive packaging and slogans that make these products interesting depending on age group, lifestyle (as UPF for vegans) and other specific groups (as people with coeliac disease or lactose intolerant) - Hyperpalatablefood with modified taste, consistency, and color to enhance flavor. - Addictive taste that makes it difficult to stop eating them once started. - Satisfies short-term hunger. - Relatively inexpensive since these are produced on a high scale and have long-shelf life. - Convenient since, due to the long shelf life, they reduce the need of trips to buy food. - Microbiologically safe. |
| --- |
